# TRIM21‐Mediated K11‐Linked Ubiquitination of ID1 Suppresses Tumorigenesis and Promotes Cuproptosis in Esophageal Squamous Cell Carcinoma

**DOI:** 10.1002/advs.202502501

**Published:** 2025-07-13

**Authors:** Lei Li, Yuqing Wang, Ruoxi Tian, Tianshuo Yang, Yaxin Liu, Baoen Shan, Lianmei Zhao

**Affiliations:** ^1^ Research Center the Fourth Hospital of Hebei Medical University Jiankang Road 12 Shijiazhuang Hebei 050011 China; ^2^ Hebei Medical University Clinical Medicine Postdoctoral Research Station Jiankang Road 12 Shijiazhuang Hebei 050011 China; ^3^ Department of Colorectal Surgery National Cancer Center/National Clinical Research Center for Cancer/ Cancer Hospital Chinese Academy of Medical Sciences and Peking Union Medical College Beijing 100021 China

**Keywords:** E3 ligase, SLC31A1, Sorafenib, TCF12, tumorigenesis

## Abstract

Inhibitor of DNA binding 1 (ID1) is a key transcriptional regulator involved in the development of various cancers, including esophageal squamous cell carcinoma (ESCC). However, the mechanisms regulating ID1 ubiquitination in ESCC are not well understood. This study identifies TRIM21 as a novel E3 ubiquitin ligase that targets ID1 for K11‐linked ubiquitination at lysine 91. Unlike typical ubiquitination that marks proteins for degradation, TRIM21‐mediated K11 ubiquitination does not affect ID1 stability. Instead, it disrupts the ID1‐TCF12 interaction, releasing TCF12 to activate SLC31A1, a copper transporter. Elevated SLC31A1 expression increases intracellular copper, inducing cuproptosis and inhibiting ESCC cell proliferation and tumor growth. Functional assays show that overexpressing TRIM21 suppresses ESCC progression, while TRIM21 knockdown promotes growth. Clinically, low TRIM21 expression correlates with advanced disease stage and poorer patient survival rate, underscoring its prognostic value. Additionally, Sorafenib treatment upregulates TRIM21, enhancing ID1 ubiquitination, increasing SLC31A1 expression, and inducing cuproptosis. These findings uncover the TRIM21‐ID1‐TCF12‐SLC31A1 axis as a critical pathway in ESCC progression, suggesting that targeting this axis with Sorafenib can offer a promising therapeutic strategy for inhibiting tumor growth and improving patient outcomes.

## Graphical abstract

ID1 is regulated by E3 ligase TRIM21, which adds non‐degradative K11‐linked ubiquitin at Lys91, disrupting ID1‐TCF12 binding and freeing TCF12 to upregulate copper transporter SLC31A1. Enhanced SLC31A1 elevates intracellular copper, triggers cuproptosis, and inhibits ESCC growth. TRIM21 expression correlates with prognosis and is induced by Sorafenib, highlighting this axis as a therapeutic target.

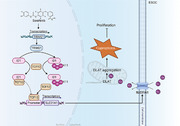

## 1 Introduction

Esophageal squamous cell carcinoma (ESCC) is a significant global health burden, with considerable geographic disparities in incidence and mortality rates. In 2020, ≈604 000 new cases and 544 000 deaths were reported globally, with ESCC constituting the majority of these cases.^[^ ^1^ ^]^ The highest incidence rates occur in East Asia, particularly China, which accounts for over half of the global burden. Despite advances in diagnosis and treatment, ESCC prognosis remains poor, with a global 5‐year survival rate below 20%, primarily due to late‐stage detection.^[^ ^2^ ^]^ Furthermore, projections indicate that by 2040, the global burden of esophageal cancer will rise significantly, reaching 987 723 new cases and 914 304 deaths, representing a 63.5% and 68.0% increase, respectively.^[^ ^3^ ^]^ These alarming trends underscore not only the urgent need for improved public health strategies and early detection programs but also the importance of advancing research into the molecular mechanisms underlying ESCC, which could lead to novel preventive and therapeutic interventions.

Inhibitor of DNA binding 1 (ID1) is a member of the basic helix‐loop‐helix (bHLH) protein family. It functions primarily as a transcriptional regulator by forming heterodimers with other bHLH proteins such as E2A, E47, and Twist.^[^ ^4^, ^5^, ^6^ ^]^ Unlike other bHLH proteins, ID1 lacks a DNA‐binding domain and therefore cannot bind directly to DNA.^[^ ^7^ ^]^ Instead, it modulates gene expression indirectly by inhibiting the DNA‐binding activities of other transcription factors. This inhibition prevents the transcriptional activation of genes involved in various aspects of cancer biology. It promotes tumor cell proliferation by regulating cell cycle proteins and accelerating G1/S phase progression.^[^ ^8^ ^]^ Additionally, ID1 is associated with cancer stemness, maintaining the self‐renewal properties of cancer stem cells by modulating signaling pathways like WNT/SHH signaling.^[^ ^9^ ^]^ Moreover, ID1 prevents apoptosis by repressing pro‐apoptotic genes like p21, contributing to resistance to chemotherapy and radiation.^[^ ^10^ ^]^ Elevated ID1 expression has been observed in various cancers, including glioblastoma, lung, breast, and prostate, where it is associated with poor prognosis and more aggressive disease stages.^[^ ^9^, ^11^, ^12^ ^]^ In our previous research, we identified elevated ID1 expression in ESCC, with high levels associated with advanced tumor stages.^[^ ^13^ ^]^ Therefore, targeting ID1 holds potential as a therapeutic strategy to inhibit tumor progression and resistance to treatments.

Ubiquitination is a crucial post‐translational modification that regulates a wide range of cellular processes by attaching ubiquitin molecules to target proteins.^[^ ^14^ ^]^ This modification is carried out through a cascade of enzymes: ubiquitin‐activating enzymes (E1), ubiquitin‐conjugating enzymes (E2), and ubiquitin ligases (E3).^[^ ^15^ ^]^ The functional outcome of ubiquitination is determined by the type and length of the polyubiquitin chain formed. K48‐linked polyubiquitin chains primarily signal protein degradation via the 26S proteasome, while K63‐linked chains are involved in DNA repair and various signaling pathways.^[^ ^16^ ^]^ Non‐canonical polyubiquitin chains, such as those linked through K6, K11, K27, K29, and K33, are less understood. In our previous study, we discovered that USP8, a deubiquitinase, stabilizes ID1 by removing its ubiquitin chains, thereby promoting the growth of ESCC.^[^ ^13^ ^]^ This finding highlights the essential role of ubiquitination in the development of ESCC and suggests the existence of a specific E3 ubiquitin ligase that facilitates ID1 ubiquitination. However, research on these specific E3 ligases in ESCC remains sparse. Understanding these ubiquitin‐mediated regulatory mechanisms is vital for developing novel therapeutic strategies for ESCC and other cancers.

In this research, we have identified TRIM21 as a highly effective E3 ubiquitin ligase targeting ID1, playing a crucial role in its K11‐linked ubiquitination. TRIM21 enhances the ubiquitination of ID1 and disrupts its interaction with TCF12, thereby inhibiting ESCC tumorigenesis and promoting cuproptosis via transcriptional upregulation of SLC31A1. Analysis of ESCC tumor samples revealed that reduced TRIM21 expression is associated with poorer patient prognosis. Importantly, we discovered that Sorafenib treatment increases the transcription of TRIM21, thereby suppressing the growth of ESCC tumors.

## 2 Results

### 2.1 TRIM21 Promotes ID1 K11‐Linked Ubiquitination

**Figure 1:**
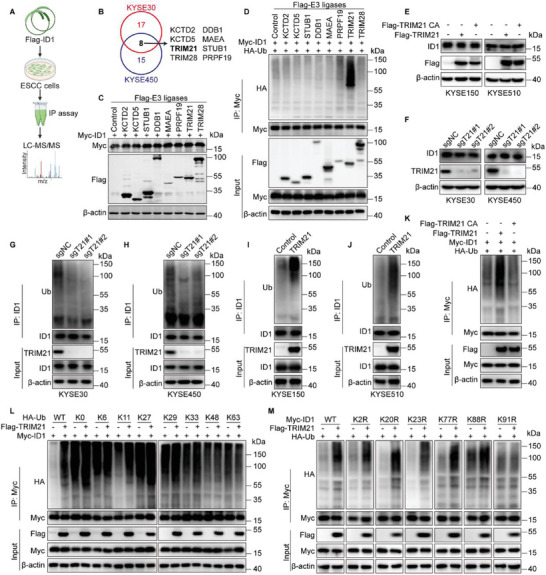
TRIM21 promotes ID1 K11‐linked ubiquitination. A) A schematic overview illustrates the immunoprecipitation‐based screen used to identify E3 ligases that are potentially associated with ID1. B) Venn diagrams show the overlap of potential E3 ligases interacting with ID1 in KYSE30 and KYSE450 cells. C) The indicated Flag‐E3 ligases and Myc‐ID1 were co‐transfected into HEK293T cells, and immunoblots were performed to assess ID1 protein levels. D) The indicated Flag‐E3 ligases, HA‐Ub, and Myc‐ID1 were co‐transfected into HEK293T cells. The ubiquitination level of ID1 was detected using an anti‐HA antibody. E) Immunoblot analysis of ID1 in KYSE150 and KYSE510 cells overexpressing TRIM21 or its CA mutant. F) ID1 protein levels were monitored by immunoblot in TRIM21‐depleted KYSE30 and KYSE450 cells. G,H) Evaluation of ID1 ubiquitination in TRIM21‐knockdown KYSE30 (G) and KYSE450 (H) cells. I,J) Assessment of ID1 ubiquitination in TRIM21‐overexpressing KYSE150 (I) and KYSE510 (J) cells. K) HA‐Ub and Myc‐ID1 were co‐expressed with TRIM21 or TRIM21 CA in HEK293T cells. The ubiquitination level of ID1 was detected using an anti‐HA antibody. L) A set of HA‐tagged ubiquitin mutants (WT, K0, K6, K11, K27, K29, K33, K48, or K63; all other lysines mutated to arginines) was co‐expressed with Myc‐ID1 and Flag‐TRIM21 in HEK293T cells, followed by anti‐HA immunoblot to determine the chain linkage pattern on ID1. M) HA‐Ub, Flag‐TRIM21 and various Myc‐ID1 mutants (WT, K2, K20, K23, K77, K88, K91; each designated lysine replaced individually) into HEK293T cells. The ubiquitination level of ID1 was detected with an anti‐HA antibody. All data from a representative experiment in at least two replicates.

To identify potential E3 ligases targeting ID1 in ESCC, we performed an immunoprecipitation (IP) experiment followed by mass spectrometry (MS) as shown in **Figure** **1**A. The MS analysis revealed eight E3 ligases interacting with ID1, including KCTD2, KCTD5, TRIM21, TRIM28, DDB1, MAEA, STUB1, and PRPF19 (Figure 1B; Figure S1A,B, Supporting Information). Since the primary function of E3 ligases is to facilitate substrate degradation via the proteasome pathway, we first assessed the protein levels of ID1 following overexpression of candidate E3 ligases. The results showed that none of the ligases affected the protein expression of ID1 (Figure 1C). However, we observed a significant increase in the ubiquitination level of ID1 upon TRIM21 overexpression (Figure 1D). To further elucidate TRIM21's impact on ID1 protein levels in ESCC, we overexpressed TRIM21 in KYSE150 and KYSE510 cells which have low endogenous TRIM21, and deleted TRIM21 in KYSE30 and KYSE450 cells which exhibit high endogenous TRIM21 (Figure S1C, Supporting Information). Consistently, neither overexpression nor knockdown of TRIM21 altered the protein levels of ID1 in ESCC cells (Figure 1E–F). Moreover, TRIM21 depletion did not affect the stability of endogenous ID1 protein (Figure S1D, Supporting Information). Notably, TRIM21 knockdown significantly reduced the ubiquitination of ID1 (Figure 1G–H), while overexpression produced the opposite effect (Figure 1I–J). In addition, the catalytically inactive TRIM21 mutant (TRIM21 CA, C16A/C31A/H33W ^[^ ^17^ ^]^) was significantly less effective than wild‐type TRIM21 in upregulating the ubiquitination level of ID1 (Figure 1K). To investigate why TRIM21 enhances ID1 ubiquitination but does not promote its protein degradation, we next examined the specific type of ubiquitin chain on ID1 affected by TRIM21. Consistent with previous results, TRIM21 specifically promoted the K11‐linked polyubiquitylation of ID1, but not other types, particularly K48‐linked ubiquitination, which typically targets proteins for proteasomal degradation (Figure 1L). To identify the key lysine residue in ID1 targeted by TRIM21 for ubiquitination, we individually mutated six lysine residues in the ID1 protein to arginine and conducted a ubiquitination assay. The results revealed that only the mutation of K91R significantly diminished the ubiquitination mediated by TRIM21, while mutations at the other five lysine residues had no such effect (Figure 1M). These results demonstrate that TRIM21 promotes the attachment of non‐canonical K11‐linked polyubiquitin chains to ID1 and highlights K91 as a critical residue for TRIM21‐mediated ubiquitination of ID1.

### 2.2 TRIM21 directly Interacts with ID1

**Figure 2:**
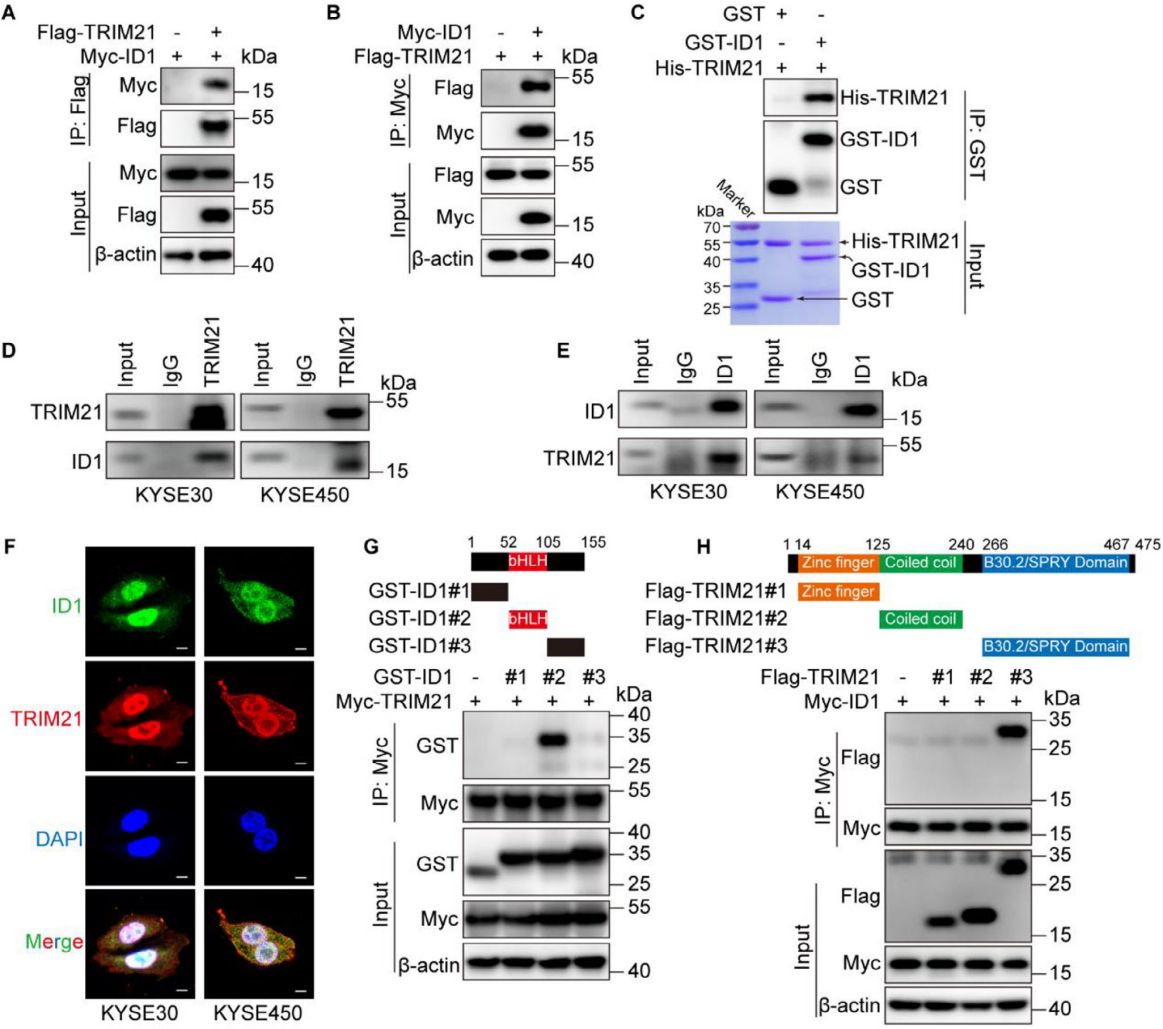
TRIM21 interacts with ID1. A) Myc‐ID1 was co‐transfected with Flag‐TRIM21 or vector into HEK293T cells, and the interaction between ID1 and TRIM21 was assessed by immunoprecipitation. B) Flag‐TRIM21 was co‐transfected with Myc‐ID1 or vector into HEK293T cells, and the ID1‐TRIM21 interaction was again verified by immunoprecipitation. C) A GST pull‐down assay confirmed the ID1‐TRIM21 interaction in vitro. D,E) The ID1‐TRIM21 association was examined by immunoprecipitation using either an anti‐TRIM21 (D) or anti‐ID1 (E) antibody. F) Endogenous TRIM21 (red) and ID1 (green) were detected by immunofluorescence in KYSE30 and KYSE450 cells. Nuclei (blue) were stained with 4,6‐diamidino‐2‐phenylindole (DAPI). Scale bars, 100 µm. G) A schematic diagram of ID1 and its truncated constructs is shown. Their ability to interact with TRIM21 was evaluated by immunoprecipitation. H) A schematic diagram of TRIM21 and its truncated constructs is presented. Their interaction with ID1 was also evaluated by immunoprecipitation.

TRIM21, as an E3 ligase, is presumed to directly interact with ID1 to regulate its ubiquitination. To investigate this, we co‐transfected Flag‐TRIM21 and Myc‐ID1 into HEK293T cells and conducted IP assays, along with GST‐pulldown experiments using recombinant TRIM21 and ID1 proteins in vitro. The results confirmed a direct interaction between TRIM21 and ID1, as TRIM21 co‐immunoprecipitated with ID1 both in cell‐based and in vitro systems (**Figure** **2**A–C). Similarly, this interaction was observed in ESCC cell lines KYSE30 and KYSE450, where TRIM21 and ID1 were successfully co‐immunoprecipitated (Figure 2D,E). Confocal microscopy further confirmed their nuclear colocalization in ESCC cells, providing additional evidence of their interaction (Figure 2F).

To determine which regions of ID1 are critical for its interaction with TRIM21, we created three truncated ID1 mutants: ID1#1 (N‐terminal region), ID1#2 (basic helix‐loop‐helix (bHLH) domain), and ID1#3 (C‐terminal region). Co‐immunoprecipitation assays revealed that the interaction with TRIM21 was specifically mediated by the bHLH domain of ID1 (Figure 2G). For TRIM21, we similarly generated three truncated mutants: TRIM21#1 (zinc finger domain), TRIM21#2 (coiled‐coil domain), and TRIM21#3 (B30.2/SPRY domain). Only the mutant containing the B30.2/SPRY domain could bind to ID1 (Figure 2H). These results suggest that the direct interaction between ID1 and TRIM21 is facilitated by the bHLH domain of ID1 and the B30.2/SPRY domain of TRIM21.

### 2.3 TRIM21 Inhibits ESCC Tumorigenesis via Ubiquitination of ID1

**Figure 3:**
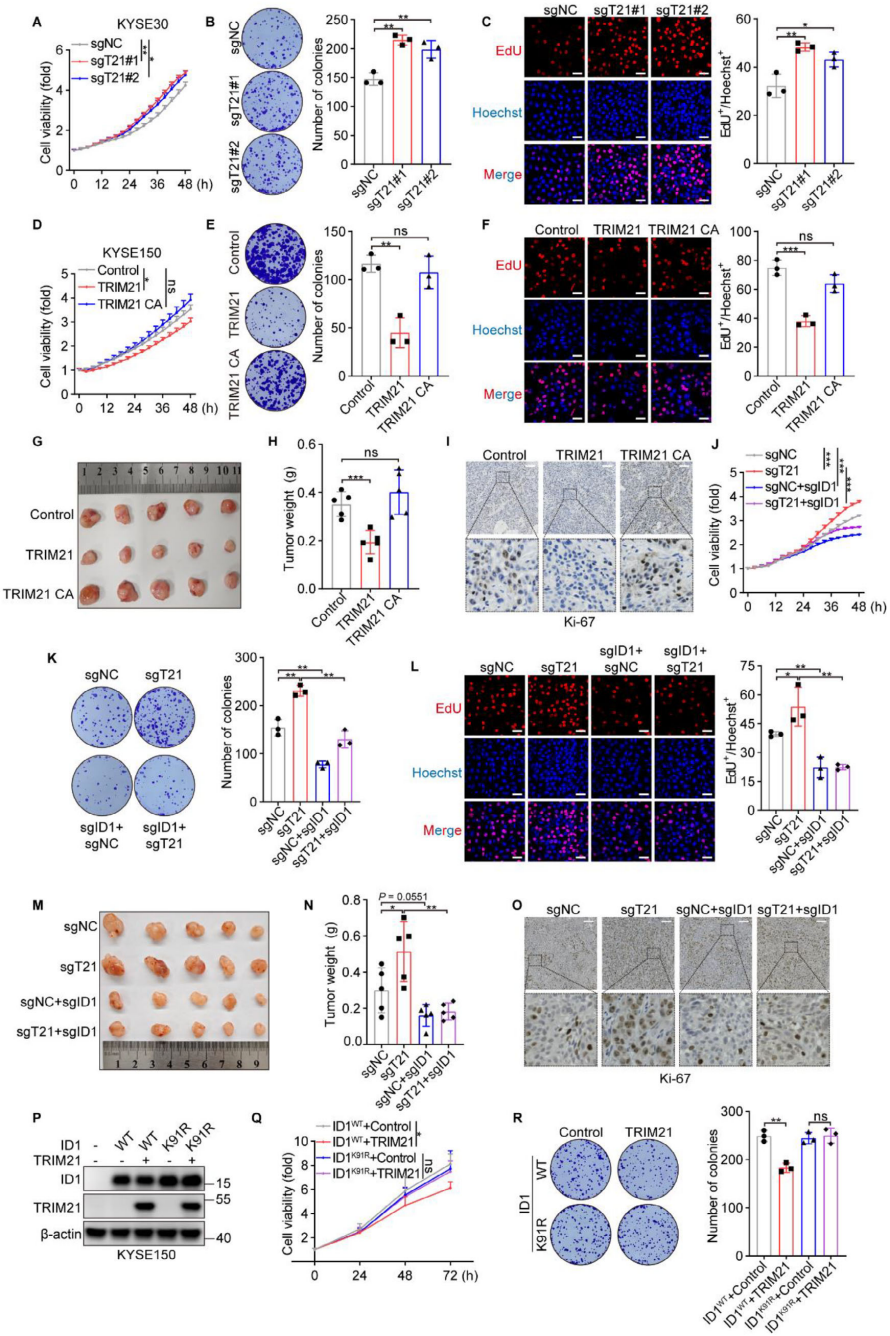
TRIM21 inhibits ESCC tumorigenesis via ubiquitination of ID1. A) The viability of the indicated KYSE30 cells was monitored using the IncuCyte live‐cell analysis system, with TRIM21‐knockdown referred to as sgTRIM21 (sgT21). B) Colony formation assays and corresponding quantitative analyses were performed in TRIM21‐knockdown KYSE30 cells. C) Representative images and the percentage of EdU‐positive cells in TRIM21‐knockdown KYSE30 cells. Scale bars, 50 µm. D–F) The cell viability, colony formation capacity, and proportion of EdU‐positive cells in KYSE150 cells overexpressing TRIM21 or TRIM21 CA. Scale bars, 50 µm. G–I) Representative tumor images (G) and tumor weights (H) of xenografts derived from KYSE150 cells overexpressing TRIM21 or TRIM21 CA. Ki67 expression (I) was assessed by immunohistochemistry. Scale bars, 50 µm. J–L) ID1 was knocked down in TRIM21‐knockdown KYSE30 cells, followed by IncuCyte analysis, colony formation assays, and EdU staining. Scale bars, 50 µm. M–O) Representative xenograft images (M) and tumor weights (N) of the indicated KYSE30 cells. Ki67 expression (O) was evaluated by immunohistochemistry. Scale bars, 50 µm. P–R) In ID1‐knockdown KYSE150 cells, either wild‐type or K91R mutant ID1 was introduced, followed by TRIM21 overexpression or vector control (P). Cell viability was measured by CCK‐8 (Q), and colony formation assays (R) were performed. ^*^ *p <* 0.05, ^**^ *p <* 0.01, ^***^ *p <* 0.001, ns, not significant (*p >* 0.05).

Given the crucial role of ID1 in ESCC tumorigenesis, we sought to determine whether TRIM21 also influences ESCC progression. To investigate this, we knocked down TRIM21 in KYSE30 and KYSE450 cells and overexpressed either wild‐type TRIM21 or its catalytically inactive mutant, TRIM21 CA, in KYSE150 and KYSE510 cells. Functional assays, including cell viability, colony formation, and 5‐ethynyl‐2′‐deoxyuridine (EdU) staining, revealed that loss of TRIM21 significantly enhanced the proliferation of ESCC cells (**Figure** **3**A–C; Figure S2A–C, Supporting Information). In contrast, overexpression of wild‐type TRIM21 markedly suppressed cell growth, whereas the TRIM21 CA mutant failed to produce a similar effect (Figure 3D–F; Figure S2D–F, Supporting Information). To validate these results in vivo, we conducted xenograft experiments using KYSE150 cells expressing control, wild‐type TRIM21, or TRIM21 CA. Tumors derived from TRIM21‐overexpressing cells exhibited significantly slower growth compared to those from control or TRIM21 CA‐overexpressing cells (Figure 3G,H). Additionally, immunohistochemical analysis showed decreased expression of the proliferation markers Ki67 in tumors with wild‐type TRIM21 overexpression (Figure 3I), further supporting its role as a suppressor of ESCC progression.

To determine whether the tumor suppressive effects of TRIM21 are dependent on ID1, we performed ID1 knockdown in both control and TRIM21‐deleted KYSE30 cells (Figure S2G, Supporting Information). Strikingly, the enhanced proliferation observed in TRIM21‐deficient cells was fully reversed upon ID1 depletion, both in vitro and in xenograft models, indicating that ID1 is essential for the regulatory function of TRIM21 (Figure 3J–O). To further validate this mechanism, we used KYSE150 cells with ID1 deletion (Figure S2H, Supporting Information) and reintroduced either wild‐type ID1 or a K91R mutant, where lysine 91 was replaced with arginine (Figure 3P). Proliferation assays revealed that TRIM21 significantly suppressed cell growth only in cells expressing wild‐type ID1, whereas the K91R mutant‐expressing cells were unaffected (Figure 3Q–R). These findings provide strong evidence that TRIM21 exerts its tumor‐suppressive effects in ESCC by targeting ID1 for regulation, specifically through the critical K91 residue.

### 2.4 TRIM21 Promotes Cuproptosis via Disrupting ID1‐TCF12 Interaction

**Figure 4:**
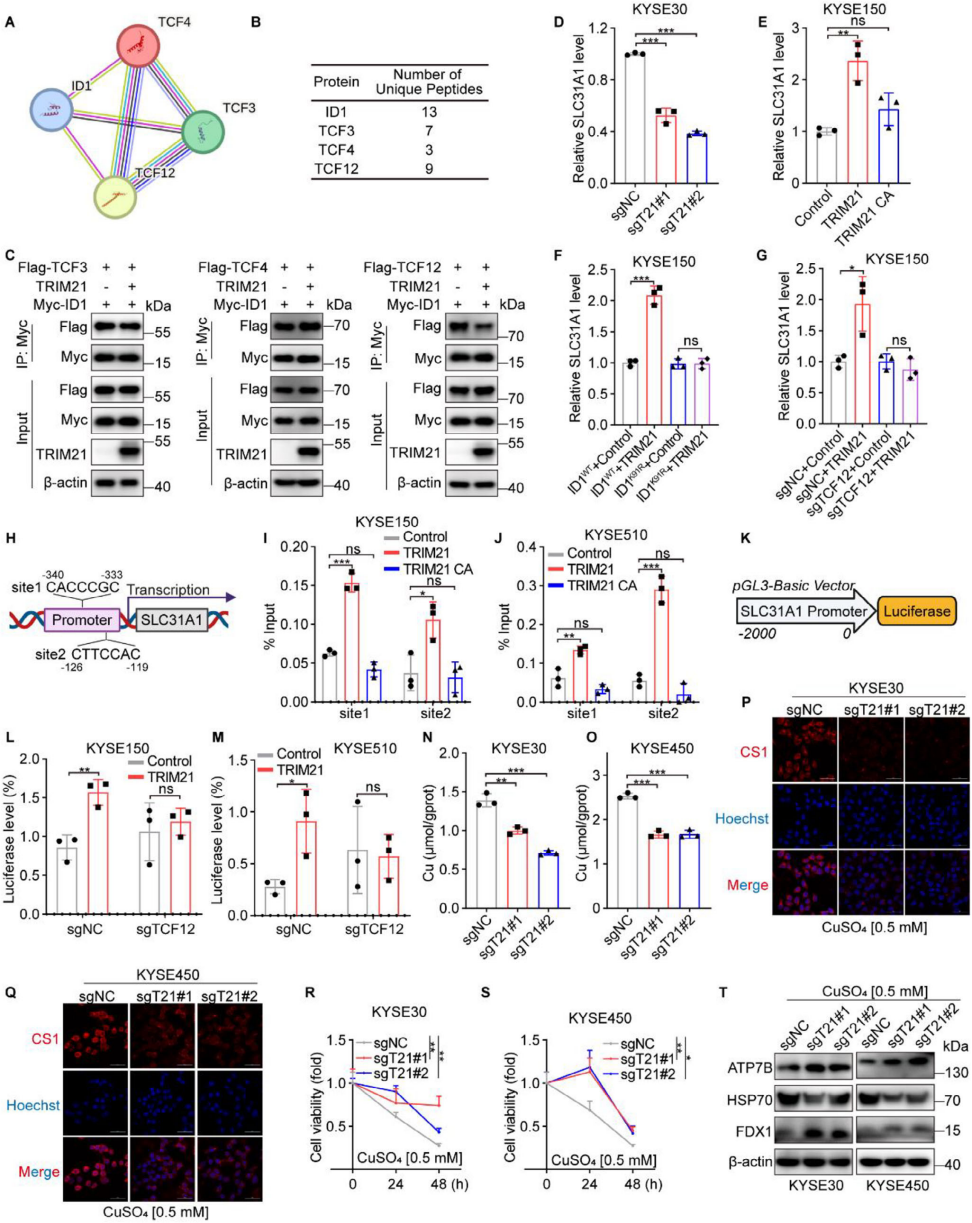
TRIM21 promotes cuproptosis via disrupting ID1‐TCF12 interaction. A) Diagram illustrating potential ID1‐interacting proteins predicted by the STRING database. B) Identification of candidate ID1‐binding proteins through mass spectrometry analysis. C) Immunoprecipitation experiments reveal interactions between Myc‐ID1 and Flag‐tagged TCF3, TCF4, or TCF12 in HEK293T cells co‐transfected with TRIM21 or control vector. D–G) Expression analysis of SLC31A1 mRNA levels across different experimental conditions. H) Illustration of TCF12 binding sites within the promoter region of SLC31A1. I,J) ChIP assays demonstrate TCF12 binding to the SLC31A1 promoter in KYSE150 (I) and KYSE510 (J) cells, with or without TRIM21 overexpression. K) Design of a luciferase reporter plasmid containing the promoter region of SLC31A1. L,M) Luciferase reporter assays in KYSE150 (L) and KYSE510 (M) cells show TRIM21‐dependent activation of the SLC31A1 promoter, even in TCF12‐knockdown conditions. N,O) Measurement of divalent copper ion concentrations in TRIM21‐knockdown KYSE30 (N) and KYSE450 (O) cells after 0.5 mM CuSO_4_ treatment. P,Q) Analysis of monovalent copper ion levels in TRIM21‐knockdown KYSE30 (P) and KYSE450 (Q) cells following 0.5 mm CuSO_4_ treatment. R,S) Cell viability assays in TRIM21‐knockdown KYSE30 (R) and KYSE450 (S) cells treated with 0.5 mm CuSO_4_. T) The protein levels of ATP7B, HSP70, and FDX1 in TRIM21‐knockdown KYSE30 cells and KYSE450 cells treated with 0.5 mm CuSO_4_. ^*^ *p <* 0.05, ^**^ *p <* 0.01, ^***^ *p <* 0.001, ns, not significant (*p >* 0.05).

Given that ID1 lacks a basic DNA‐binding domain, it typically inhibits bHLH transcription factors by forming heterodimers, thereby blocking their DNA‐binding ability and transcriptional activity. We hypothesized that TRIM21‐mediated ubiquitination of ID1 might influence its interaction with bHLH factors, leading to the suppression of ESCC growth. To test this hypothesis, we explored potential ID1‐binding partners using the STRING database, which predicted interactions with three bHLH transcription factors: TCF3, TCF4, and TCF12 (**Figure** **4**A). Interestingly, these same interactions were independently identified in previous mass spectrometry analysis, further supporting that ID1 associates with TCF3, TCF4, and TCF12 (Figure 4B). To assess whether TRIM21 affects these interactions, we co‐transfected Myc‐ID1 with each bHLH factor, along with either control or TRIM21, into HEK293T cells and performed an IP assay. As shown in Figure 4C, TRIM21 overexpression specifically reduced the interaction between ID1 and TCF12, while the interactions with TCF3 and TCF4 were unaffected. Furthermore, ZDOCK molecular docking revealed that Lys91 of ID1 forms a hydrogen bond with Glu577 of TCF12 (Figure S3A, Supporting Information). Importantly, mutation of ID1 at Lys91 abrogated TRIM21's ability to disrupt the ID1‐TCF12 interaction (Figure S3B, Supporting Information). These results suggest that TRIM21‐mediated ubiquitination selectively regulates the interaction between ID1 and TCF12.

It has been reported that ID1 degradation frees TCF12, enabling it to promote the transcription of SLC31A1.^[^ ^18^ ^]^ To explore whether TRIM21 regulates SLC31A1 expression by modulating the interaction between ID1 and TCF12, we performed a series of experiments. We found that TRIM21 knockdown significantly reduced SLC31A1 mRNA levels in ESCC cells, while overexpression of TRIM21 enhanced them (Figure 4D,E; Figure S3C,D, Supporting Information). Importantly, this effect was dependent on wild‐type ID1, as cells expressing the K91R ID1 mutant showed no change in SLC31A1 levels upon TRIM21 overexpression (Figure 4F). Likewise, in TCF12‐deleted cells (Figure S3E, Supporting Information), TRIM21 failed to upregulate SLC31A1, highlighting the essential role of TCF12 in this process (Figure 4G; Figure S3F, Supporting Information). To investigate the underlying mechanism, we conducted chromatin immunoprecipitation (ChIP) experiments, which revealed that TRIM21 overexpression significantly increased TCF12 binding to the SLC31A1 promoter region (Figure 4H–J). Moreover, dual‐luciferase reporter assays demonstrated that TRIM21 enhanced SLC31A1 transcription only when TCF12 was present (Figure 4K–M). These results indicate that TRIM21 promotes SLC31A1 transcription by releasing TCF12 from its inhibitory interaction with ID1, thereby activating its transcriptional activity on the SLC31A1 promoter.

SLC31A1 is a transmembrane protein responsible for transporting copper ions into cells and plays a key role in regulating cuproptosis.^[^ ^19^ ^]^ Based on this, we hypothesized that TRIM21 may promote copper ion accumulation in ESCC cells by enhancing SLC31A1 transcription, thereby triggering cuproptosis. To test this hypothesis, we first investigated how changes in TRIM21 expression affect copper ion levels in ESCC cells. Knockdown of TRIM21 significantly reduced the copper uptake capacity of KYSE30 and KYSE450 cells (Figure 4N–Q). Furthermore, TRIM21‐deficient cells displayed greater tolerance to additional copper ion treatment compared to control cells (Figure 4R,S). We next analyzed the expression of cuproptosis‐related markers and observed a significant decrease in the levels of HSP70, along with a notable increase in ATP7B and FDX1, in TRIM21‐knockdown cells (Figure 4T). Moreover, TRIM21 knockdown significantly suppressed the aggregation of dihydrolipoamide S‐acetyltransferase (DLAT), a key hallmark of copper‐induced cell death (Figure S3G, Supporting Information). These findings suggest that TRIM21 promotes cuproptosis by upregulating SLC31A1 expression, highlighting its potential role in regulating copper ion metabolism and cell death pathways in ESCC.

### 2.5 Reduced TRIM21 Serves as a Poor Prognostic Indicator in ESCC

**Figure 5:**
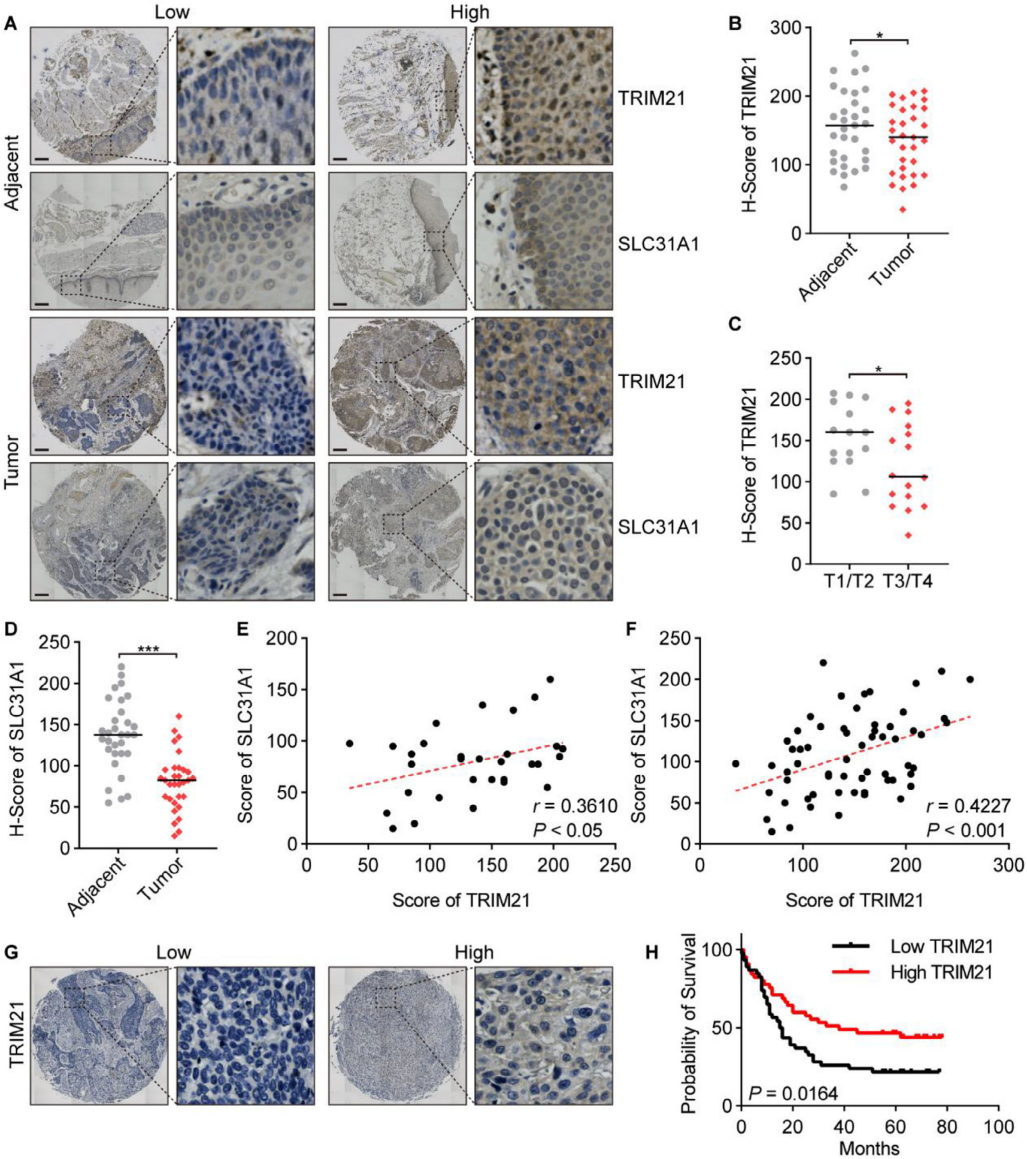
TRIM21 downregulation serves as a positive prognostic indicator in ESCC. A) Representative IHC images showing the expression of TRIM21 and SLC31A1 in 31 paired ESCC and adjacent normal tissue samples. Scale bars: 50 µm. B) Comparative analysis of TRIM21 expression scores between tumor tissues and their corresponding adjacent normal tissues. C) Assessment of the correlation between TRIM21 expression scores and clinical T stage. D) Comparative analysis of SLC31A1 expression scores between tumor tissues and adjacent normal tissues. E) Evaluation of the correlation between TRIM21 and SLC31A1 expression levels within tumor tissues. F) Evaluation of the correlation between TRIM21 and SLC31A1 expression levels across both tumor tissues and adjacent normal tissues. G) Representative IHC images showing the expression of TRIM21 in 91 ESCC tissue samples. Scale bars: 50 µm. H) Kaplan‐Meier plot of the overall survival of ESCC patients stratified by TRIM21 expression. ^*^ *p <* 0.05, ^***^ *p <* 0.001.

To establish the clinical relevance of TRIM21 in ESCC progression, we evaluated its expression patterns and prognostic value in patient specimens. Immunohistochemical analysis revealed significant downregulation of TRIM21 in ESCC tumor tissues compared with matched adjacent normal epithelia (**Figure** **5**A,B). Notably, progressive depletion of TRIM21 protein expression correlated with advanced pathological staging (Figure 5C), suggesting a tumor stage‐dependent suppression pattern. Consistent with its proposed role in regulating SLC31A1 expression, parallel immunohistochemical staining demonstrated concomitant reduction of SLC31A1 in malignant lesions relative to normal mucosal controls (Figure 5A,D). Quantitative analysis using Spearman correlation revealed a strong positive linear relationship between TRIM21 and SLC31A1 expression levels across both neoplastic and histologically normal tissue specimens (Figure 5E,F). Finally, survival analysis of 91 ESCC patients showed that low TRIM21 expression was significantly associated with poorer overall survival (Figure 5G,H).

### 2.6 Sorafenib Suppresses ESCC Tumorigenesis by Upregulating TRIM21

**Figure 6:**
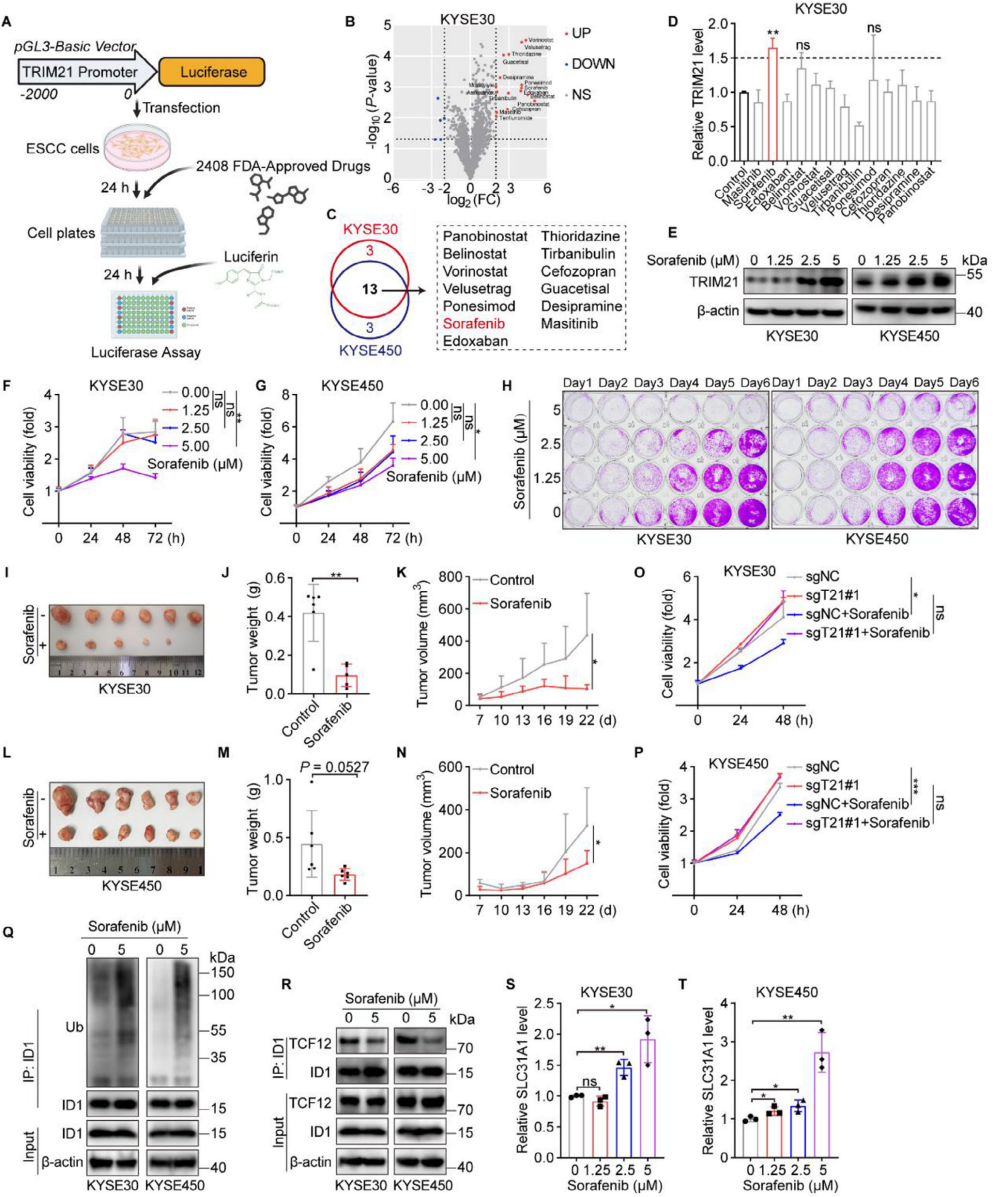
Sorafenib suppresses ESCC tumorigenesis by upregulating TRIM21. A) Schematic representation of the drug screening strategy designed to identify compounds that regulate TRIM21 transcription. B) Compounds that markedly modulate TRIM21 transcription (|log2 (FC)| > 2 and *p* < 0.05) in KYSE30 cells. C) Venn diagrams showing the common and unique candidate compounds identified in both cell lines. D) The mRNA level of TRIM21 in KYSE30 cells treated with indicated drugs. E) The protein levels of TRIM21 in KYSE30 and KYSE450 cells treated with increasing concentrations of Sorafenib (0, 1.25, 2.5, 5 µm). F–H) Assessment of KYSE30 and KYSE450 cell viability following Sorafenib treatment using CCK‐8 assays (F and G) and SRB staining (H). I–N) Tumorigenicity assays in BALB/c nude mice subcutaneously injected with KYSE30 (I–K) or KYSE450 (L–N) cells, followed by treatment with Sorafenib (50 mg kg^−1^) or corn oil (vehicle control). Tumor volumes and weights were measured and analyzed. O,P) CCK‐8 assay evaluating Sorafenib's effect on the viability of KYSE30 (O) and KYSE450 (P) cells following TRIM21 knockdown. Q) Evaluation of ID1 ubiquitination in KYSE30 and KYSE450 cells treated with 5 µm Sorafenib. R) Immunoprecipitation analysis of the interaction between TCF12 and ID1 in KYSE30 and KYSE450 cells treated with 5 µm Sorafenib. S,T) The mRNA level of SLC31A1 in KYSE30 (S) and KYSE450 (T) cells treated with different concentrations of Sorafenib (0, 1.25, 2.5, 5 µm). ^*^ *p <* 0.05, ^**^ *p <* 0.01, ^***^ *p <* 0.001, ns, not significant (*p >* 0.05).

Recognizing TRIM21 as a tumor suppressor, we sought to identify potential drugs that could enhance its transcription using a library of 2408 FDA‐approved and marketed compounds. A luciferase reporter assay was employed for the screening (**Figure** **6**A), resulting in the identification of 16 candidate drugs that elevated TRIM21 transcription in KYSE30 and KYSE450 cells, with 13 showing consistent effects across both cell lines (Figure 6B,C; Figure S4A, Supporting Information). Among these, Sorafenib emerged as the most promising compound, significantly increasing TRIM21 expression in both cell lines during subsequent validation (Figure 6D; Figure S4B, Supporting Information). In addition, the protein level of TRIM21 was increased by Sorafenib in a dose‐dependent manner (Figure 6E). To investigate whether Sorafenib could inhibit ESCC progression, we performed a series of in vitro and in vivo studies. Sorafenib treatment significantly reduced the proliferative ability of ESCC cells in vitro and effectively suppressed tumor growth in xenograft models (Figure 6F–N). To confirm whether Sorafenib's anti‐tumor activity in ESCC depends specifically on TRIM21, we performed rescue experiments using both in vitro CCK‐8 proliferation assays and in vivo subcutaneous xenograft models. In control cells, Sorafenib treatment markedly inhibited cell proliferation in vitro and suppressed tumor growth in vivo, whereas these effects were significantly blunted in TRIM21‐knockdown cells (Figure 6O‐P; Figure S4C,D, Supporting Information).

Further mechanistic analysis revealed that Sorafenib treatment enhanced ID1 ubiquitination in ESCC cells (Figure 6Q), disrupting its interaction with TCF12 (Figure 6R) and subsequently promoting the expression of SLC31A1 (Figure 6S‐T). Additionally, Sorafenib increased intracellular copper ion levels and activated the expression of genes associated with cuproptosis pathways (Figure S4E–G, Supporting Information). Notably, TRIM21 knockdown significantly attenuated the ability of Sorafenib to induce cuproptosis in ESCC cells (Figure S4H–J, Supporting Information). These findings suggest that Sorafenib exerts potent anti‐ESCC therapeutic effects by upregulating TRIM21 expression, highlighting its potential as a novel treatment strategy for ESCC.

## 3 Discussion

**Figure 7:**
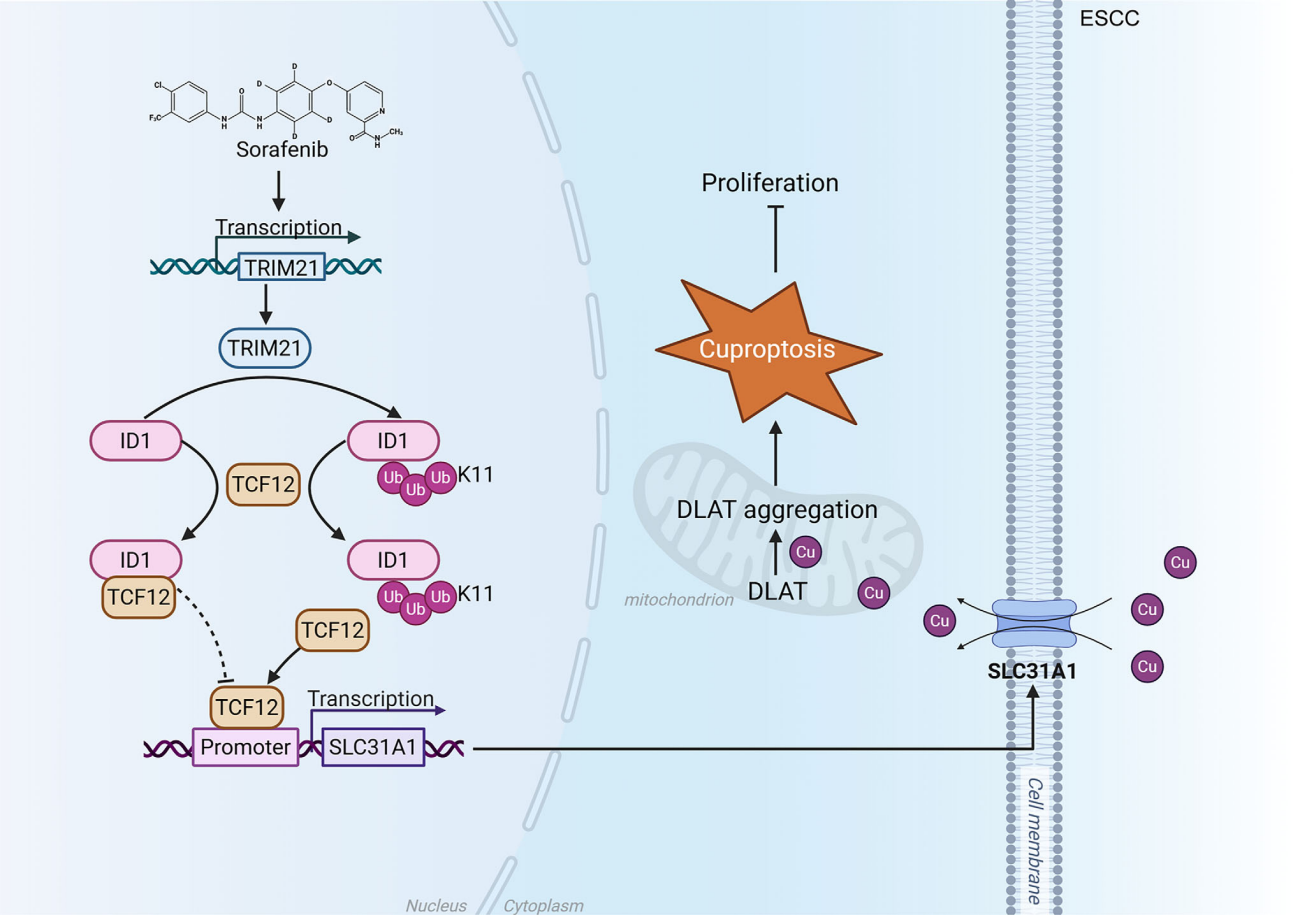
Schematic diagram of this study. The proposed model illustrates how Sorafenib enhances TRIM21 transcription, a key mechanism in suppressing tumorigenesis and promoting cuproptosis in ESCC. TRIM21 facilitates the ubiquitination of ID1, disrupting its interaction with TCF12. This disruption frees TCF12, enabling it to activate SLC31A1 transcription, which increases copper ion uptake into the cell. This pathway highlights the pivotal role of TRIM21 in linking Sorafenib treatment to enhanced copper‐dependent cell death. The figure was created with BioRender.com.Schematic diagram of this study. The proposed model illustrates how Sorafenib enhances TRIM21 transcription, a key mechanism in suppressing tumorigenesis and promoting cuproptosis in ESCC. TRIM21 facilitates the ubiquitination of ID1, disrupting its interaction with TCF12. This disruption frees TCF12, enabling it to activate SLC31A1 transcription, which increases copper ion uptake into the cell. This pathway highlights the pivotal role of TRIM21 in linking Sorafenib treatment to enhanced copper‐dependent cell death. The figure was created with BioRender.com.

ID1 functions as a pivotal transcriptional regulator implicated in various human cancers, including ESCC. It inhibits the transcriptional activity of TCF12 on the SLC31A1 gene by forming a heterodimer with TCF12.^[^ ^20^ ^]^ Previous research has demonstrated that the E3 ubiquitin ligases Cdh1 and Smurf2 facilitate the K48‐linked ubiquitination and subsequent degradation of ID1.^[^ ^21^, ^22^ ^]^ In our study, we have identified TRIM21 as a robust E3 ligase for ID1, which promotes K11‐linked ubiquitination of ID1 and disrupts its interaction with TCF12. This disruption liberates TCF12, allowing it to activate the transcription of SLC31A1. TRIM21 promotes SLC31A1 expression by enhancing ID1 ubiquitination, thereby inhibiting tumor growth and inducing cuproptosis via the TRIM21‐ID1‐TCF12‐SLC31A1 axis (**Figure** **7**). Additionally, low levels of TRIM21 expression are associated with poor prognosis in ESCC patients. Importantly, our findings indicate that treatment with Sorafenib effectively suppresses ESCC tumor growth by upregulating TRIM21 transcription, suggesting that Sorafenib may offer a superior therapeutic option for certain patients with ESCC.

TRIM21 is a standout member of the tripartite motif (TRIM) protein family, which is defined by a conserved motif consisting of a RING finger domain, one or two B‐box domains, and a coiled‐coil region.^[^ ^23^ ^]^ Its broad substrate specificity underpins its diverse roles in cellular regulation, immune responses, and disease pathogenesis.^[^ ^24^ ^]^ By mediating the degradation of pivotal regulatory proteins such as p53 and IRF3, TRIM21 has been linked to both cancer progression and antiviral defense.^[^ ^25^, ^26^ ^]^ Additionally, it exerts control over apoptosis and ferroptosis by modulating the ubiquitination levels of FSP1, NCAPH, and GPX4.^[^ ^27^, ^28^, ^29^ ^]^ Here, we show that TRIM21 promotes K11‐linked ubiquitination of ID1 without affecting its overall protein stability. Instead, TRIM21 inhibits the proliferation of ESCC cells by blocking ID1 from binding to TCF12. As a result, TCF12 is liberated to enhance the transcription of the copper transporter SLC31A1, thereby driving cuproptosis and potentially offering a novel therapeutic avenue.

Sorafenib is a multi‐kinase inhibitor originally approved for advanced hepatocellular carcinoma (HCC) and renal cell carcinoma (RCC).^[^ ^30^, ^31^ ^]^ By targeting kinases such as VEGFR, PDGFR, and RAF, it exerts broad antitumor effects.^[^ ^32^ ^]^ Beyond these canonical targets, accumulating studies indicate that Sorafenib can trigger diverse cell death pathways: it lowers glutathione (GSH) to induce ferroptosis, and it elevates autophagy‐related proteins (e.g., Beclin‐1, LC3‐II) to promote autophagosome formation.^[^ ^33^, ^34^ ^]^ In this investigation, we found that Sorafenib markedly inhibits ESCC tumorigenesis and progression by enhancing TRIM21 transcription. Furthermore, Sorafenib boosts the ubiquitination of ID1 while disrupting its interaction with TCF12, thereby upregulating SLC31A1 and inducing cuproptosis. Taken together, these findings underscore Sorafenib's potential as a pivotal therapeutic option for ESCC.

## 4 Experimental Section

### Cell Culture

The human ESCC cell lines, including KYSE450, KYSE30, KYSE150, and KYSE510, were cultured in RPMI‐1640 medium (Thermo Fisher Scientific, USA) supplemented with 10% fetal bovine serum (FBS; LONSERA, USA). HEK293T cells were maintained in DMEM medium (Thermo Fisher Scientific, USA) containing 10% FBS. All cell lines were incubated at 37 °C in a humidified atmosphere with 5% CO_2_.

### Plasmids, Reagents and Antibodies

The following plasmids were constructed: Flag‐tagged E3 ligases, Flag‐TCF3, Flag‐TCF4, Flag‐TCF12, and Myc‐ID1, along with TRIM21 truncated mutants, were cloned into the pcDNA3 vector. TRIM21 and its mutant form TRIM21 CA (C16A/C31A/H33W) were generated in the PLVX vector. Single guide RNAs (sgRNAs) targeting ID1, TRIM21, and TCF12 were cloned into the pLentiCrisprV2 vector. The promoters of SLC31A1 and TRIM21 were inserted into the pGL3‐basic vector, and ID1 truncated mutants were cloned into the pEBG‐GST vector. All primers for plasmid construction and sgRNA target sequences are provided in Table S1 (Supporting Information).

Reagents used in this study include: MG132 (Sigma‐Aldrich, USA), cupric sulfate (MedChemExpress, USA), sorafenib (MedChemExpress, USA), Protein A/G PLUS‐Agarose (Santa Cruz Biotechnology, USA), Anti‐c‐Myc Magnetic Beads (MedChemExpress, USA), Pierce Quantitative BCA Protein Assay Kit (Thermo Fisher Scientific, USA), TRIzol Reagent (Invitrogen, USA), puromycin (Sangon Biotech, China), G418 (Sigma‐Aldrich, USA), and 4,6‐diamidino‐2‐phenylindole (DAPI; Sangon Biotech, China).

Antibodies used were as follows: anti‐TRIM21 (PA5‐22294, Invitrogen, USA), anti‐HA (3724S, Cell Signaling Technology, USA), anti‐Flag (14793S, Cell Signaling Technology, USA), anti‐ubiquitin (#43 124, Cell Signaling Technology, USA), anti‐TCF12 (14419‐1‐AP, Proteintech, China), anti‐MYC (16286‐1‐AP, Proteintech, China), anti‐β‐actin (6609‐1‐Ig, Proteintech, China), anti‐KI67 (27309‐1‐AP, Proteintech, China), goat anti‐rabbit IgG (AS014, ABclonal, China), goat anti‐mouse IgG (AS003, Proteintech, China), anti‐HSP70 (sc‐24, Santa Cruz Biotechnology, USA), anti‐ATP7A (sc‐376467, Santa Cruz Biotechnology, USA), anti‐GST (sc‐138, Santa Cruz Biotechnology, USA), anti‐ID1 (sc‐133104, Santa Cruz Biotechnology, USA), and anti‐FDX1 (12592‐1‐AP, Proteintech, China).

### Cell Viability Assay

Cells were seeded in 96‐well plates at a density of 2000 cells per well. After 24 h of incubation, the culture medium was replaced with 200 µL of fresh RPMI‐1640 medium. For the IncuCyte S3 Live‐Cell Analysis System, cell images were acquired every 3 h. Following 72 h of continuous monitoring, the cell numbers were quantified, and proliferation rates were calculated based on the recorded data. For the CCK‐8 assay, 10 µL of CCK‐8 reagent was added to each well at 0, 24, 48, and 72 h. After 1 h of incubation at 37 °C, the absorbance of each well was measured at 450 nm using a microplate reader. Cell proliferation rates were then calculated based on the optical density values.

### Colony Formation Assay

Cells (500 per well) were seeded in 6‐well plates with RPMI‐1640 medium, replaced every 3 days. After 10 days, cells were washed with PBS, fixed with 4% paraformaldehyde (15 min), and stained with 1% crystal violet (15 min). Plates were washed, air‐dried, and photographed. Colonies (> 50 cells) were counted.

### EdU Staining

The indicated cells were seeded into a µ‐Slide VI chamber (ibidi, Germany) and allowed to recover overnight. The EdU staining was performed using Click‐iT EdU Imaging Kits (C10338, Invitrogen, USA) following the manufacturer's instructions. Cells were incubated with 10 µm EdU for 2 h, fixed with 3.7% formaldehyde, permeabilized with 0.5% Triton X‐100, and blocked with 3% BSA in PBS. A Click‐iT reaction cocktail was applied for 30 min at room temperature, followed by staining with Hoechst 33342 for 30 min. Imaging was performed using a laser confocal microscope.

### GST Pull‐Down Assay

ID1 was cloned into the pGEX‐6p‐2 vector with a GST tag and purified. TRIM21‐His protein (18010‐H07B, Sino Biological, China) was incubated with either GST‐ID1 or GST protein at a 3:1 molar ratio in lysis buffer. The mixture was immunoprecipitated with anti‐GST antibody at 4 °C overnight, followed by incubation with Protein A/G beads for 4 h. After extensive washing with lysis buffer, bound proteins were eluted and analyzed by immunoblotting. GST fusion proteins were verified by Coomassie Brilliant Blue staining.

### Immunoprecipitation and Ubiquitination Assay

For the immunoprecipitation assay, cells were treated with MG132 (20 µm) for 6 h to inhibit proteasomal activity and then lysed in lysis buffer. The resulting lysates were incubated overnight at 4 °C with specific primary antibodies and protein A/G magnetic beads. After incubation, the beads were washed three times with lysis buffer to remove non‐specific proteins, and the immunoprecipitated complexes were analyzed by immunoblotting using the appropriate antibodies.

For the ubiquitination assay, the initial steps were identical to the immunoprecipitation protocol. However, to evaluate the ubiquitination of ID1, the purified complexes were specifically probed with an anti‐ubiquitin antibody during immunoblotting.

### Immunofluorescence Staining

The target cells were cultured in a µ‐Slide VI chamber (ibidi, Germany). Subsequently, the cells were fixed with 4% paraformaldehyde and permeabilized using Triton X‐100. To minimize non‐specific antibody binding, the cells were blocked with 5% bovine serum albumin (BSA). Primary antibody incubation was carried out overnight at 4 °C. The next day, the cells were treated with secondary antibodies conjugated to Alexa Fluor 488 (#4416, Cell Signaling Technology, USA) for mouse IgG and Alexa Fluor 555 (#4409, Cell Signaling Technology, USA) for rabbit IgG. DAPI was used to stain the nuclei, and imaging was performed with a laser confocal microscope.

### Immunohistochemical Staining

Immunohistochemistry was performed using the immunoperoxidase method. Tissue sections were first deparaffinized in xylene and rehydrated through an alcohol series. Antigen retrieval was carried out, followed by blocking with goat serum albumin for 1 h at room temperature. The sections were then incubated with primary antibodies overnight at 4 °C. Afterward, the sections were treated with secondary antibodies, and the signals were developed with diaminobenzidine and counterstained using hematoxylin. The slides were subsequently scanned using the TissueFAXS Viewer (TissueGnostics, Austria). The staining intensity was scored on a scale of 0 (no staining), 1 (weak staining), 2 (moderate staining), and 3 (strong staining). Human ESCC tissue samples and their paired adjacent normal tissues were collected from the Fourth Hospital of Hebei Medical University with ethical approval from the hospital's committee and informed consent from all patients.

### Quantitative Real‐Time PCR (qRT‐PCR)

Total RNA was isolated from the specified cells using TRIzol reagent (Thermo Scientific, USA) and subsequently converted into cDNA with the Quantscript RT Kit (Applied Biological Materials, Canada) as per the provided protocol. Quantitative real‐time PCR (qRT‐PCR) was carried out using GoTaq qPCR Master Mix (Promega, USA) following the manufacturer's guidelines. Details of the primers used for qRT‐PCR are provided in Table S2 (Supporting Information).

### Mass Spectrometry (MS)

KYSE30 and KYSE450 cells transfected with control or ID1‐Flag plasmids were harvested 48 h post‐transfection. Immunoprecipitated samples were separated by SDS‐PAGE, and gel slices were subjected to in‐gel digestion with trypsin. Peptides were extracted with 0.1% formic acid (FA), desalted using a C18 column, and freeze‐dried. The peptide concentration was determined using a BCA kit. Finally, 1 µg of peptides was loaded onto an Easy‐nLC1200 system, and proteomic analysis was conducted with a Q Exactive HF mass spectrometer.

### Dual‐Luciferase Assay

The SLC31A1 promoter was inserted into the pGL3‐basic luciferase reporter vector. Both the luciferase reporter and the Renilla luciferase plasmids were co‐transfected into the specified cells. Luciferase activity was measured using the Dual‐Luciferase Reporter Assay System (E1910, Promega, USA) according to the manufacturer's protocol. The results were normalized against the Renilla luciferase signal for accurate analysis.

### Chromatin Immunoprecipitation (ChIP)

The ChIP assays were performed using the SimpleChIP Enzymatic Chromatin IP Kit (9003S, Cell Signaling Technology, USA), following the manufacturer's protocol. The binding of the TCF12 protein to the SLC31A1 promoter was analyzed via qRT‐PCR. Primer sequences used for the ChIP assay are listed in Table S2 (Supporting Information).

### Copper Detection Assays

Intracellular divalent copper ions were quantified using the Copper Colorimetric Assay Kit (E‐BC‐K300‐M, Elabscience, China) following the manufacturer's instructions. For monovalent copper ion detection, cells were seeded into a µ‐Slide VI chamber (ibidi, Germany) and stained with 5 µm Coppersensor‐1 (HY‐141511, MedChemExpress, USA) at 37 °C for 10 min in the dark. Nuclei were counterstained with Hoechst 33342, and imaging was conducted using a laser confocal microscope.

### Sulforhodamine B (SRB) Staining

Cells were seeded into 24‐well plates at a density of 5000 cells per well, with six wells prepared for each group and one well collected daily. After washing with PBS, the cells were fixed with 10% TCA for 30 min and then rewashed. On the final day, the cells were air‐dried for 1 h, stained with 0.4% SRB (230 162, Sigma‐Aldrich, USA) for 20 min, and washed three times with 1% acetic acid. Once dried, images were captured and analyzed.

### Drug Screening

The TRIM21 promoter was cloned into the pGL3‐basic luciferase reporter vector. The reporter plasmid was then transfected into KYSE30 or KYSE450 cells. Following a 24‐h treatment with a library of 2408 FDA‐approved drugs (MedChemExpress, USA), luciferase activity was assessed using the One‐Lumi Firefly Luciferase Reporter Gene Assay Kit (RG055M, Beyotime, China) following the manufacturer's instructions. Drugs exhibiting |log2 (FC)| > 2 and *p* < 0.05 were selected, and common hits between the two cell lines were identified.

### Animal Studies

For the subcutaneous tumor xenograft assay, 1 × 10⁶ cells were subcutaneously injected into 6‐week‐old male BALB per c nude mice. Tumors were harvested three weeks post‐injection. In the Sorafenib therapeutic experiment, mice were treated daily with Sorafenib (50 mg kg^−1^) or corn oil (vehicle control) via oral gavage for two weeks. Tumor size was measured every three days, and tumor volume was calculated using the formula: length × width^2^ / 2. Tumors were collected three weeks after injection. The BALB/c nude mice were obtained from Spafu Biotechnology Co., Ltd. (Beijing) and housed at the Experimental Animal Center of the Fourth Hospital of Hebei Medical University. All animal experiments were approved by the Animal Protection Committee of the Fourth Hospital of Hebei Medical University (No. 2 023 049) and were conducted in strict accordance with relevant animal welfare guidelines.

### Statistics

All data are expressed as mean ± SD, unless otherwise noted, and were subjected to unpaired *t*‐tests for comparison. *Pearson*'s correlation coefficient was used to assess the relationship between TRIM21 and SLC31A1 in tissue samples. The expression levels of TRIM21 and SLC31A1 in ESCC versus normal tissues were compared using paired *t*‐tests. Survival data were analyzed with the Kaplan‐Meier method, and differences were assessed by a two‐sided log‐rank test. Statistical analyses were performed using GraphPad Prism 8 software. A *p*‐value of < 0.05 was considered statistically significant. Significance levels are denoted as ^*^ *p* < 0.05, ^**^ *p* < 0.01, and ^***^ *p* < 0.001 in the figures. Results labeled as NS are not significant (*p* > 0.05).

## Conflict of Interest

The authors declare no conflict of interest.

## Author Contributions

L.L., Y.W., and R.T. contribute equally to this work. L.L. designed the research and performed most experiments. L.L. and Y.W. contributed to several experiments. R.T. performed data analysis. T.Y. and Y L. helped with the animal experiments. L.L. and Y. W. wrote the manuscript. L.Z. and B.S. supervised this project and revised the manuscript.

## Supporting information



Supporting Information

## Data Availability

The data that support the findings of this study are available in the supplementary material of this article.
