# Supplementary material for: TRIM21‐Mediated K11‐Linked Ubiquitination of ID1 Suppresses Tumorigenesis and Promotes Cuproptosis in Esophageal Squamous Cell Carcinoma
Source: Adv Sci (Weinh). 2025 Jul 13;12(35):e02501. doi: 10.1002/advs.202502501 (PMC12462935; doi:10.1002/advs.202502501)
Supplement: Supplementary file 1 — Supporting Information [file ADVS-12-e02501-s001.docx]

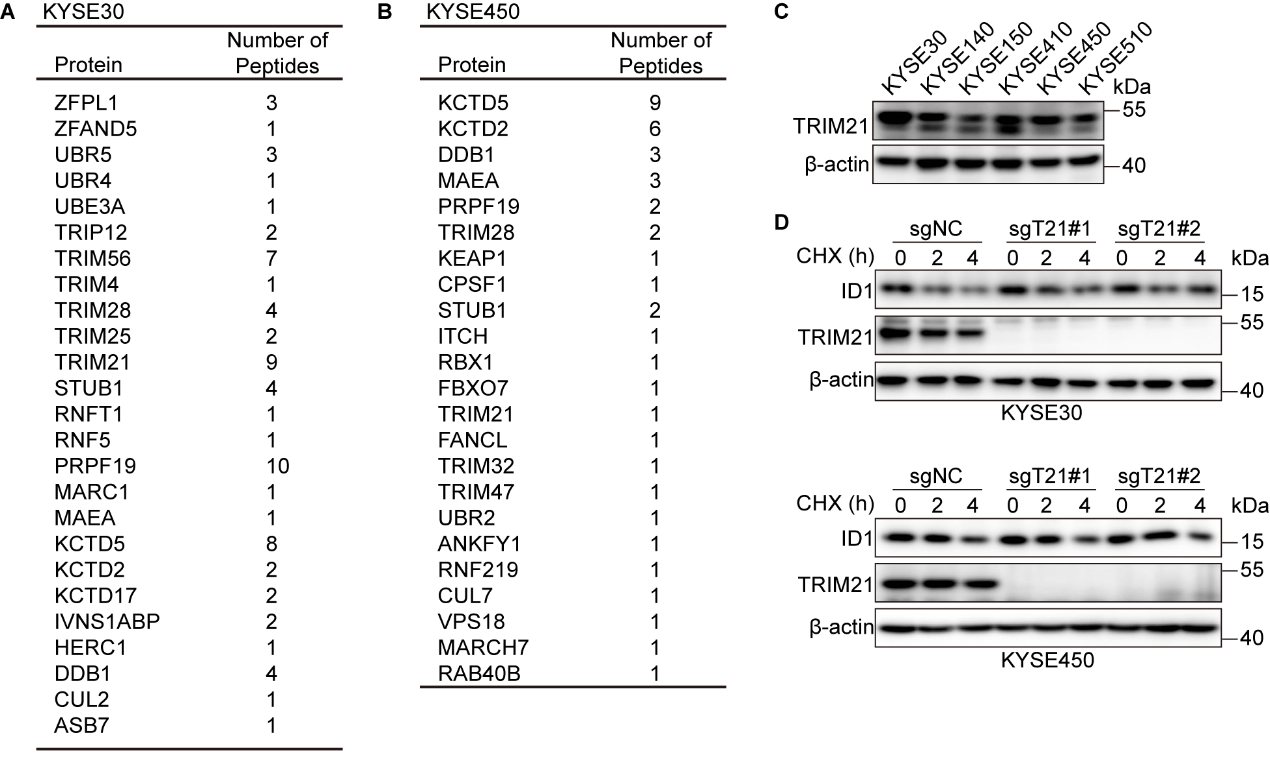
**Figure S1 TRIM21 promotes ID1 K11-linked ubiquitination**

(A-B) Identification of candidate ID1-binding ubiquitination-related proteins through mass spectrometry analysis in KYSE30 (A) and KYSE450 (B) cells. (C) The protein levels of TRIM21 in ESCC cell lines were detected by immunoblotting. (D) The degradation of ID1 in TRIM21-knockdown KYSE30 and KYSE450 cells treated with CHX (50 μg/ml) for the indicated times.


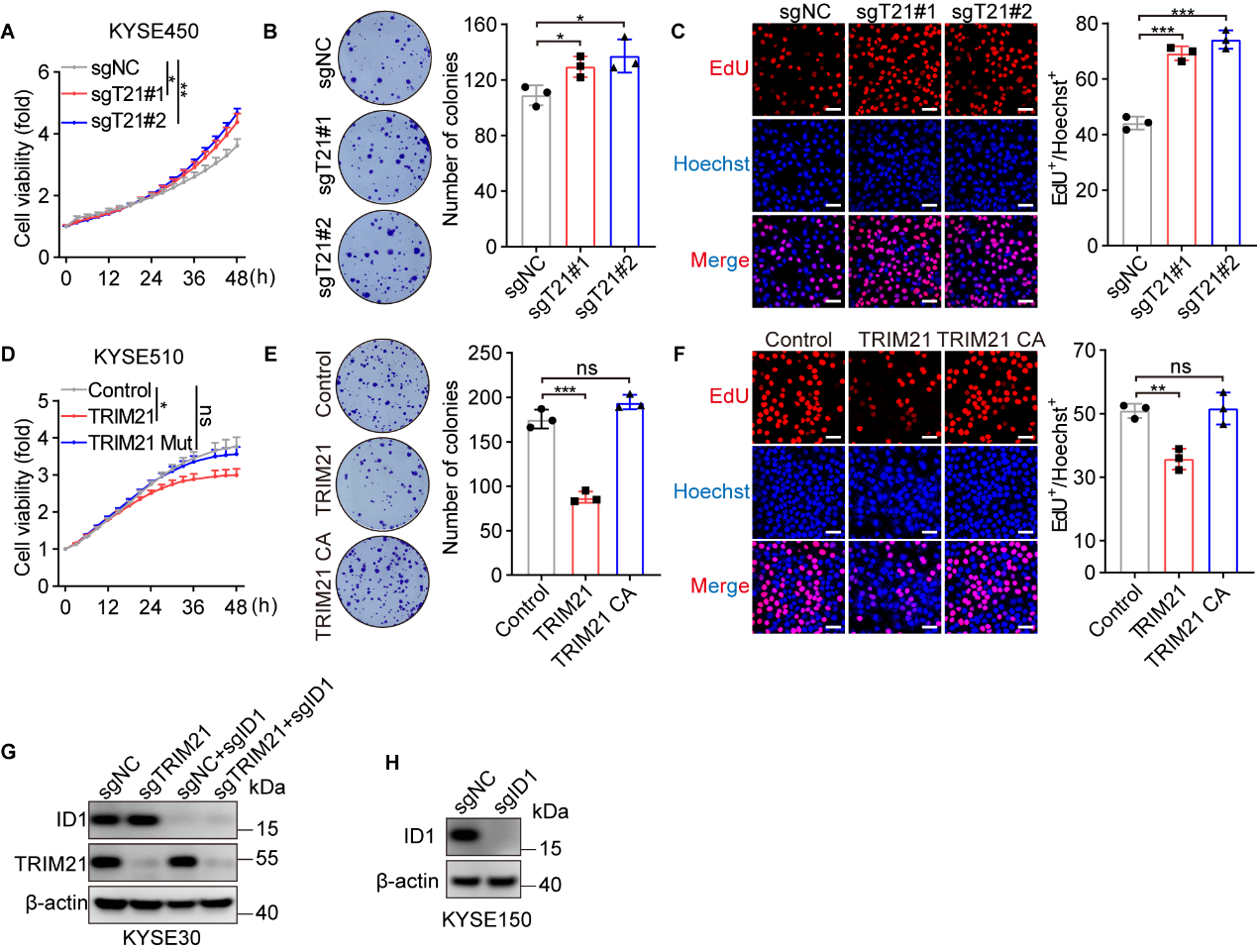
**Figure S2 TRIM21 inhibits ESCC tumorigenesis via ubiquitination of ID1**

(A-C) The cell viability, colony formation capacity, and proportion of EdU-positive cells in TRIM21-knockdown KYSE450 cells. Scale bars, 50 μm. (D-F) The cell viability, colony formation capacity, and proportion of EdU-positive cells in KYSE510 cells overexpressing TRIM21 or TRIM21 CA. Scale bars, 50 μm. (G) The protein levels of ID1 and TRIM21 in indicated KYSE30 cells. (H) The protein level of ID1 in ID1-knockdown KYSE150 cells. **P <* 0.05, ***P <* 0.01, ****P <* 0.001, ns, not significant (*P >* 0.05).


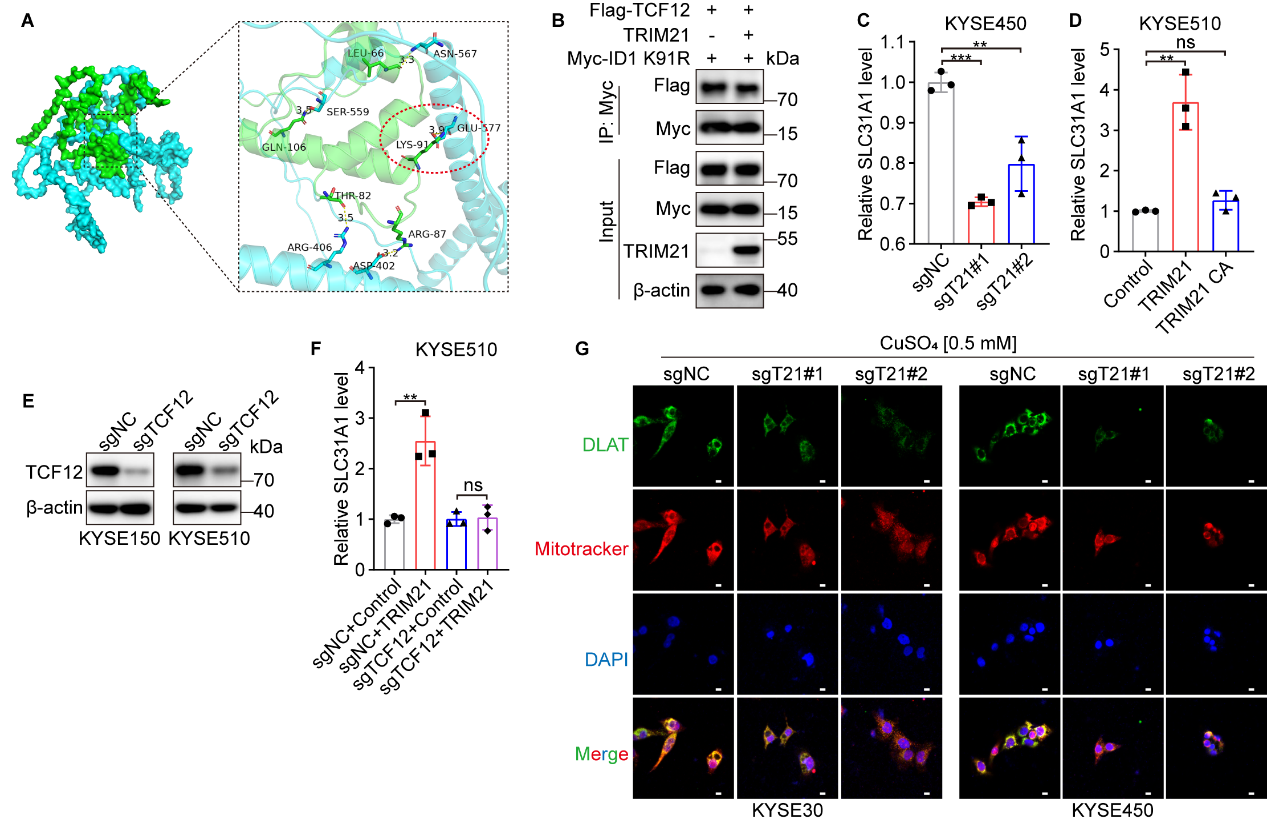
**Figure S3 TRIM21 promotes cuproptosis via disrupting ID1-TCF12 interaction**

(A) The ZDOCK molecular docking model of ID1 and TCF12. (B) Immunoprecipitation experiments reveal interactions between Myc-ID1 K91R and Flag-tagged TCF12 in HEK293T cells co-transfected with TRIM21 or control vector. (C) The mRNA level of SLC31A1 in TRIM21-knockdown (sgT21) KYSE450 cells. (D) The mRNA level of SLC31A1 in KYSE510 cells overexpressing TRIM21 or TRIM21 CA. (E) The protein level of TCF12 in TCF12-knockdown KYSE150 and KYSE510 cells. (F) The mRNA level of SLC31A1 in indicated KYSE510 cells. (G) The DLAT (green) distribution in TRIM21-knockdown KYSE30 and KYSE450 cells treated with 0.5 mM CuSO_4_ were examined by immunofluorescence. Nuclei (blue) were stained with DAPI, mitochondria were stained with mitotracker dyes (red). Scale bars, 10 μm. ***P <* 0.01, ****P <* 0.001, ns, not significant (*P >* 0.05).

**
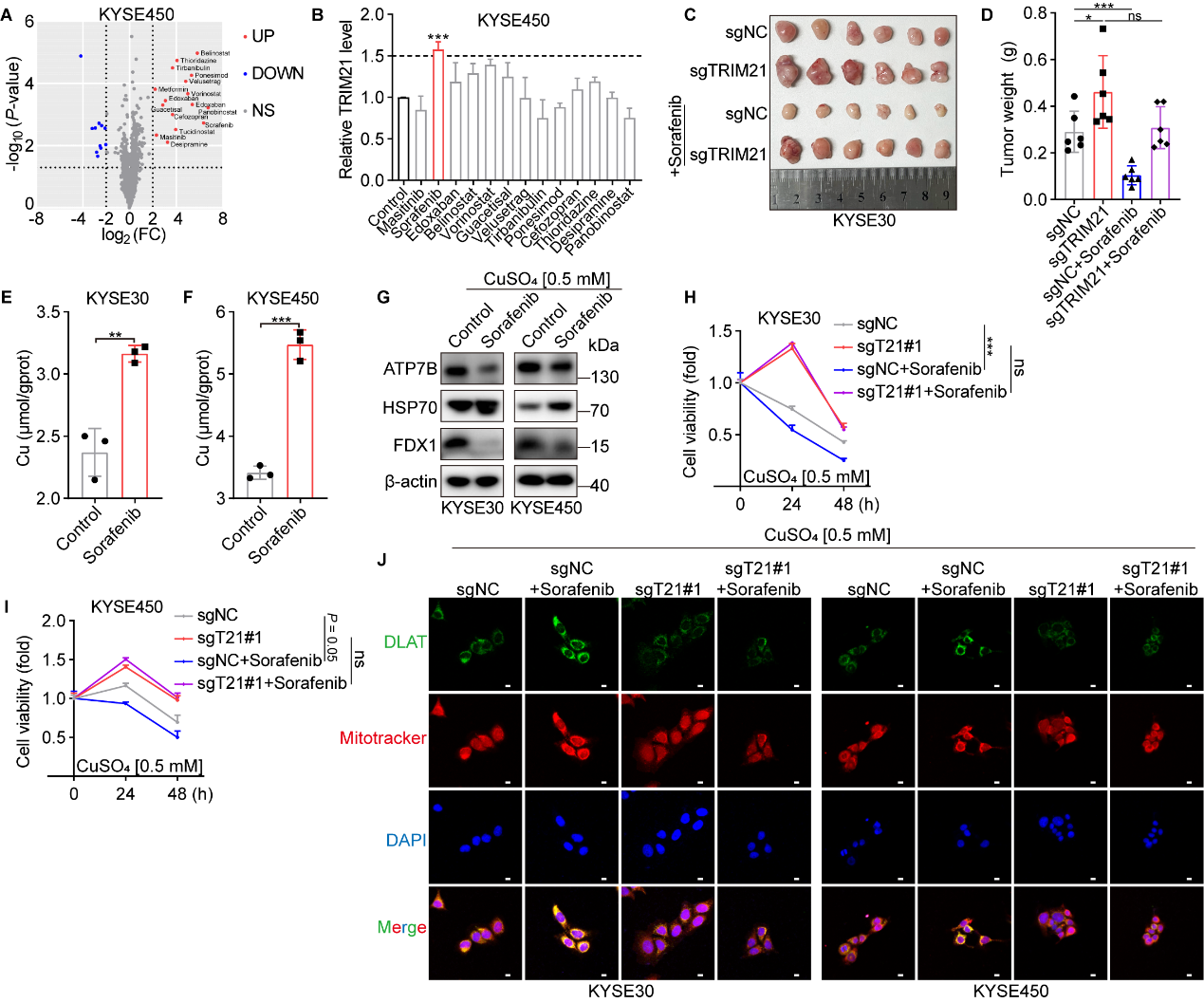
Figure S4 Sorafenib suppresses ESCC tumorigenesis by upregulating TRIM21**

(A) Compounds that markedly modulate TRIM21 transcription (|log2 (FC)| > 2 and *P* < 0.05) in KYSE450 cells. (B) The mRNA level of TRIM21 in KYSE450 cells treated with indicated drugs. (C-D) Representative tumor images (C) and tumor weights (D) of xenografts derived from control or TRIM21-knockdown KYSE30 cells treated with Sorafenib (50 mg/kg) or corn oil (vehicle control). (E-F) The monovalent copper ion levels of KYSE30 (E) and KYSE450 (F) cells treated with 5 μM Sorafenib and 0.5 mM CuSO_4_. (G) The protein levels of ATP7B, HSP70 and FDX1 in KYSE30 cells and KYSE450 cells which treated with 5 μM Sorafenib and 0.5 mM CuSO_4_. (H-I) Cell viability assays in TRIM21-knockdown KYSE30 (H) and KYSE450 (I) cells treated with 5 μM Sorafenib and 0.5 mM CuSO_4_. (J) The DLAT (green) distribution in TRIM21-knockdown KYSE30 and KYSE450 cells treated with 5 μM Sorafenib and 0.5 mM CuSO_4_ were examined by immunofluorescence. Nuclei (blue) were stained with DAPI, mitochondria were stained with mitotracker dyes (red). Scale bars, 10 μm. **P <* 0.05, ***P <* 0.01, ****P <* 0.001, ns, not significant (*P >* 0.05).

| **Table S1. Primers used for plasmids construction and target sequences of sgRNAs.** | | |  |
| --- | --- | --- | --- |
|  | Forward | Reverse |  |
| KCTD2 | ATGGCGGAACTGCAGCTGGACCCGG | CATCCGCGATCCTCTCTCCTGAAGA |  |
| KCTD5 | ATGGCGGAGAATCACTGCGAGCTCC | CATCCTTGAGCCTCGTTCTTGCAAA |  |
| TRIM28 | ATGGCGGCCTCCGCGGCGGCAGCCT | CTTGTCATCGTCATCCTTGTAGTCG |  |
| DDB1 | ATGTCGTACAACTACGTGGTAACGG | TGGATCCGAGTTAGCTCCTCCACAA |  |
| MAEA | ATGGCGGTGCAGGAGTCGGCGGCTC | CATGATGTACACCTTCTCGGCTTGT |  |
| STUB1 | ATGAAGGGCAAGGAGGAGAAGGAGG | GTAGTCCTCCACCCAGCCATTCTCA |  |
| PRPF19 | ATGTCCCTAATCTGCTCCATCTCTA | CAGGCTGTAGAACTTGAGGCTTCTG |  |
| TRIM21 | ATGGCTTCAGCAGCACGCTTGACAA | ATAGTCAGTGGATCCTTGTGATCCA |  |
| ID1 | ATGAAAGTCGCCAGTGGCAGCACCG | GCGACACAAGATGCGATCGTCCGCA |  |
| ID1#1 | ATGAAAGTCGCCAGTGGCAGCACCG | GCGCGCCCCGGCGCCCCCGGCGCAG |  |
| ID1#2 | CTGCCTGCCCTGCTGGACGAGCAGC | AAGGTCCCTGATGTAGTCGATGACG |  |
| ID1#3 | CAGTTGGAGCTGAACTCGGAATCCG | GCGACACAAGATGCGATCGTCCGCA |  |
| TRIM21#1 | ATGGTCACATGCCCTATCTGCCTGG | CATGGCGTGGTCACGGTGTTTCCGA |  |
| TRIM21#2 | ATGGTCCCTCTTGAGGAGGCTGCAC | TGCTGAGCTGTGGCACCTTCGATCT |  |
| TRIM21#3 | ATGTCTCCAGAACTCAGGAGTGTGT | AATATTCAGTGGACAGAGGGTTAGA |  |
| TCF3 | ATGAACCAGCCGCAGAGGATGGCGC | CATGTGCCCGGCGGGGTTGTGGGCT |  |
| TCF4 | ATGCATCACCAACAGCGAATGGCTG | CATCTGTCCCATGTGATTCGATGCG |  |
| TCF12 | ATGAATCCCCAGCAACAACGCATGG | CATATGACCCATAGGGTTGGTAGTT |  |
| sgTRIM21#1 | | GGACTGAAGAAGGGCCGCAG |  |
| sgTRIM21#2 | TCATCTCAGAGCTAGATCGA | / |  |
| sgID1 | AGCACGTCATCGACTACATC | / |  |
| sgTCF12 | TGACTGCTTCCCAGTGTAGT | / |  |
| **Table S2. Primers used for qRT-PCR and ChIP.** | | |  |
|  | Forward | Reverse |  |
| GAPDH | AGGGCTGCTTTTAACTCTG | CTGGAAGATGGTGATGGG |  |
| SLC31A1 | AAGGACTCAAGATAGCCCGAG | TGGGACAGGCATGGAATTGTA |  |
| SLC31A1 site1 | AACAATCCGCCGGTATCTCT | TGAAAGCCAACGCAATGTTCG |  |
| SLC31A1 site2 | ACGGTCTCTGGACCGAAAGT | CGCGCGAGCCTCTCATTT |  |
